# Emerging stronger from COVID-19 through arts

**DOI:** 10.1177/17579759221118256

**Published:** 2022-09-02

**Authors:** Chong Yau Ong, Lee Wai Ching Deanna

**Affiliations:** 1 Medicine, Newcastle University Medicine, Iskandar Puteri Johor, Malaysia; 2 Department of General Medicine, Sengkang General Hospital, Singapore

**Keywords:** COVID-19, Malaysia, public health, health promotion, arts

## Abstract

**Objectives:**

To assess art initiatives and programmes communicating health messages and during the COVID-19 pandemic in Malaysia from 2020 to 2021.

**Study design:**

Qualitative narrative study.

**Methods:**

Grey literature on COVID-19 art initiatives and programmes in Malaysia was reviewed.

**Results:**

The art initiatives and efforts in Malaysia to promote health and wellness during the COVID-19 pandemic were compelling and notable. These initiatives can be classified into five major categories, namely performing arts, visual arts, culture, literature and digital art. The main health messages conveyed through these initiatives included public education regarding COVID-19, health precautionary steps recommended for the public, and solidarity in the fight against COVID-19. These art initiatives cultivated positive responses from the public and increased their awareness of COVID-19.

**Conclusion:**

From the public health perspective, the use of arts to educate and create awareness of COVID-19 in Malaysia were encouraging. The public is made more informed and prepared to face the challenges ahead.

The arts and humanities define who we are as a people. That is their power – to remind us of what we each have to offer, and what we all have in common. To help us understand our history and imagine our future. To give us hope in the moments of struggle and to bring us together when nothing else will. (Michelle Obama) (1)

This quote summarises strongly how creative arts can impact our lives, especially in the present struggle with the COVID-19 pandemic. The World Health Organization (WHO)’s scoping review of over 900 publications in 2019 covering almost 4000 studies has shown the beneficial impact of arts in promoting health and preventing ill health (2). Arts is also a powerful tool to convey and communicate messages to the population. Art activities can involve aesthetic engagement, involvement of the imagination, sensory activation, evocation of emotion and cognitive stimulation. Such activities often include social interaction and physical activity. This could invoke psychological, physiological, social and behavioural responses associated with health outcomes, improvements in quality of life and psychological wellbeing (3).

In addition to high mortality and morbidity rates of COVID-19, the disrupted normalcy of daily lives and economic downfall during the pandemic has severely impacted the physical and mental health of the world’s population. Amid these tribulations, arts and humanities have provided an opportunity to communicate consolation and hope and have been used as an outreach tool for health promotion to the public, especially in social isolation. Even as the creative arts industry has been severely affected, it has been observed that it continues to evolve, surpassing the odds of the pandemic with rapid digitisation of the industry via virtual platforms utilisation and social media engagement. The pandemic has also been considered a source of inspiration to artists. Hence, using arts as a communication and outreach tool to promote health is more important than ever in the COVID-19 pandemic, especially in this digital age. The Centers of Disease Control and Prevention (CDC) encourages public health professionals to recognise arts and culture as valuable and assessable community resources, thereby engaging them as partners in promoting vaccine confidence and uptake (4). In this review, we examine the health messages communicated through arts in the five categories of performing arts, visual arts, literature, culture and digital arts in Malaysia during the COVID-19 pandemic (5). The types of health messages studied are specific to COVID-19, which includes but is not restricted to raising the awareness of the public, reinforcing the importance of preventive strategies and precautionary measures, promoting adherence to standard operating procedures (SOP), resilience and appreciation.

## Objectives

To assess art initiatives communicating health messages during the COVID-19 pandemic in Malaysia from 2020 to 2021.

## Study design

This is a narrative, qualitative review on the health communication and benefits through arts in Malaysia. We assessed the content of health messages and its form of delivery through the various art initiatives at the community level in Malaysia from 2020 to 2021.

## Methods

Search databases of PubMed Central and ERIC yielded 16 results (see Appendices 1 and 2). However, all were excluded due to non-inclusion of arts (*n* = 16), not having prevention component (*n* = 15) and not having been conducted in Malaysia (*n* = 1). Grey literature on COVID-19 art initiatives and programmes in Malaysia was reviewed. Health messages communicated through the art initiatives and the corresponding potential benefits were examined independently by the authors.

## Results

### Performing arts

Performing arts broadly encompasses activities in the genres of music, dance, theatre, singing and film. During the pandemic in Malaysia, performing arts gave insights and updates and delivered various health promotion messages to combat the pandemic (6).

One of the earlier health promotion messages placed emphasis on raising the awareness of the public and reinforcing the importance of adhering to standard operating procedures. This can be seen in some music composed and performed by the public – for example, in ‘Malaysia Covid-19 Song’, the composer creatively incorporated scenes from Bollywood movies and used captivating lyrics to send a message about the perils of COVID-19 and to urge the public to be adherent to lockdown measures by minimising outdoor activities and travels (7). These messages were also communicated through a song by JY Boy which encourages the public to heed the lockdown and preventative measures by government, and stand united against COVID-19 (7).

The messages to promote resilience and solidarity are also evident in the local performing arts. During the COVID-19 lockdown period, the local television media company produced a short Chinese film entitled *Bonus Vacation*. The theme of the series was based on close-to-real-life struggles of individuals and their families during the pandemic, and how they emerged stronger with positivism and solidarity. Additional health promotion messages included active, healthy living with tips on healthy food choices and indoor physical activities during the lockdown. The film won the Jury’s Special Prize at Seoul International Drama Award 2021 (8).

Other messages conveyed through performing arts are appreciation of efforts by the frontline health care workers and volunteers, and the call for togetherness. The international online streaming platform Netflix also showcased a documentary titled *Frontliner* to give honour and gratitude to healthcare workers, food delivery drivers and other individuals who worked tirelessly in this pandemic (9). This film was ranked in the top 10 on Netflix for 6 weeks. Apart from the film, the song ‘To you and me, heroes’ expressed thankfulness to frontliners and calls for togetherness in combating coronavirus (7).

### Visual arts

Visual arts refer to crafts, design, painting, photography, sculpture and textiles. Many artists embarked on portraying frontline workers to acknowledge and thank them for their efforts in curtailing COVID-19 (10,11).

Some of these paintings included public murals depicting healthcare workers in personal protective equipment tirelessly carrying their duties for the nation. Other murals contain the paintings of healthcare workers with the Director-General of Health Malaysia – hailing them for their efforts in combating COVID-19 (12). These paintings also served as a reminder to the public that the threat of COVID-19 in the community is real and to be vigilant and bear social responsibility in reducing COVID-19 transmission, especially to vulnerable groups.

**Figure 1.**
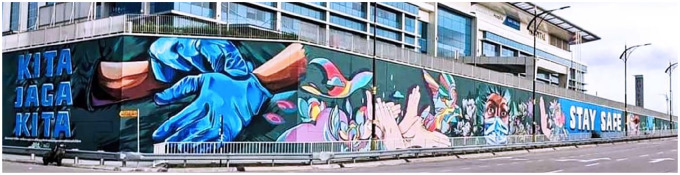
Giant mural on the walls of a private hospital communicating precautionary measures that the public can undertake.

One giant mural was painted along the walls of a private hospital overlooking a road with the slogan ‘Kita Jaga Kita’ (We take care of ourselves) (Figure 1) (13). It conveys the message of raising the awareness of the public on precautionary measures as well as paying tribute to the frontliners. This was an initiative by the hospital with the Ministry of Tourism, Arts and Culture Malaysia jointly organised by the National Disaster Management Agency.

### Culture

While COVID-19 has prevented physical gatherings and art activities in Malaysia, it certainly did not hamper the zeal of artists and art enthusiasts. Art exhibitions moved onto digital and virtual platforms where the outreach to the public proved to be stupendous. For instance, the Sabah state art gallery received more entries for its 35th Sabah Annual Art Selection than in pre-pandemic times (14). This proves to be a positive sign that people are invested even in their lockdown time towards meaningful works and art. The theme of submission was between Mount Kinabalu and New Norm. Han Hee Guan in his *Early Day at the Market* painted people wearing masks in the marketplace – a new norm that is necessary to keep the pandemic at bay. An acrylic artwork by At Tirmizie titled *Wear Mask* depicted five human figures of different ages and genders wearing face coverings. Moris Dexter Mitanis’s acrylic work on wood titled *Narrow Space* showed two men avoiding crowded places in keeping with social distancing; *Pandemic COVID-19* Story by John Austen showcased activities of public responsibilities such as not spreading fake news, going for vaccination and shopping online. In her work titled *Silent Battle*, Titi Noor showed six hands holding six wrists forming an alliance against the virus. *Suffering of COVID* by Asnedi used mix materials to show people in poverty pleading for daily necessities, and the hope that vaccines will bring.

### Literature

One of the positive impacts of lockdown is on reading habits – an increased number of uptakes and hours spent in reading (15,16). In Malaysia, although there was no study conducted on reading patterns during the pandemic, one of the largest bookstores experienced a significant increase in book sales. The National Library of Malaysia saw the borrowing of digital reading materials more than double in 2020, mainly in the fiction genre. The virtual network of the library had an almost 10-fold increase in membership from 2544 in February 2020 to 22,584 members in March 2020. Most new members were adults, followed by teenagers (17).

At the same time, the book titled *COVID-19 and Psychology in Malaysia: Psychosocial Effects, Coping and Resilience* was written to illustrate the socio-psychological experiences of Malaysians during the pandemic (18). One chapter was devoted to the ways in which Malaysians have learnt to be more resilient during this health crisis.

### Digital and electronic arts

During the pandemic, we observed the vibrancy of digital and electronic arts, with some using social media platforms. Of note, the ‘Stay Home, Stay Safe’ project created by Rtist Malaysia encourages public to upload their COVID-19-themed artwork, with part of the incentives channelled to support small to medium enterprises and frontliners (19).

Health promotion through animation videos featuring Malaysia preschool beloved characters such as Didi, Papa Pipi, and Upin and Ipin was not lacking (20–22). Among the messages conveyed were regular hand washing, staying indoors, social distancing and the importance of vaccination. These animations have garnered millions of viewings and the positive responses received have motivated the local producer (with an enormous number of subscribers worldwide) to translate them into various languages to cater for global viewers, especially in countries which remain badly affected by COVID-19.

## Discussion

At the time of writing, there were no published articles measuring specific outcomes attained by the various art efforts in Malaysia. It is indeed challenging to evaluate such efforts quantitatively or estimate the behavioural outcomes given the once-off nature of the opportunistic engagements (23). Table 1 summarises the messages and possible health benefits of art initiatives and efforts in Malaysia.

**Table 1.** Messages and possible benefits of art initiatives and programmes in Malaysia.

	Messages	Possible benefits	
		Author/producer	Audience/observer
Performing arts	- Audio-visual narration of struggle of nation during pandemic. - Coping and adaptation to new norms. - Togetherness/unity. - Resilience. - Social responsibility.	- Satisfaction from increased viewing and positive comments. - Social responsibility. - Recognition if awarded.	- Increased awareness. - Entertainment. - Relaxation. - Physical and mental wellbeing.
Visual art	- Acknowledgment and thankfulness to frontline workers. - Adherence to preventive recommendations.	- Satisfaction from completion of artwork. - Social responsibility.	- Increased awareness. - Sense of being cared for. - Social responsibility.
Culture	- Depiction of struggle of nation. - Ways to reduce transmission. - Adaptation to new norms. - Resilience. - Togetherness/unity.	- Social responsibility.	- Aesthetic gain from participation and appreciation. - Increased awareness. - Mental wellbeing.
Literacy	- Written narration of struggle of the nation. - Coping and adaptation to new norms. - Resilience.	- Increased knowledge from discussion and sharing with co-authors. - Social responsibility.	- Increased knowledge. - Mental wellbeing. - Benefits of pleasure reading.
Digital arts	- Ways to reduce transmission. - Social responsibility.	- Satisfaction from increased viewing and positive comments. - Corporate branding from collaborations. - Social responsibility.	- Increased awareness. - Entertainment.

The benefits of performing arts, especially music, in health promotion are not limited to: (a) stimulating the body into dance, physical activities and walking; (b) distracting listeners from pain towards meaningful activities; (c) reducing stress and promoting relaxation; and (d) renewal of strength to face challenges in life (24,25). In keeping with trends in other countries, the lyrics of songs composed in line with COVID-19 cover aspects of preventive strategies (26). Additionally, acknowledgement and appreciation of the frontline workers were frequently observed in the pandemic-related songs in Malaysia. Positive contents and messages in the music were uplifting for the public in times of difficulties.

As for visual arts, the public health messages conveyed were congruent with the efforts globally. Warnings of the dangers of COVID-19 were communicated; also the tireless work by healthcare workers, as well as preventive measures that the community can undertake at their capacity. The strategic placement of murals in high-traffic areas served as a platform to reach out to the wider public. It can ignite reflections and conversations among the public. This is in keeping with initiatives in other countries to create awareness and to promote COVID-19 preventive behaviours. Observing or creating community art or mural paintings can offer relief and therefore is a mechanism for coping. It also enhances community cohesion and can be utilised to create emotional safety spaces (27).

The public health themes displayed in the exhibits in Malaysia showed many similarities in the themes synthesised from a study of children’s art exhibits in India which surrounds positivity and experiences, unity, safety, hope, uncertainty, gratitude, faith and future expectations (28). The quantity and diversity of art pieces were enough to communicate the Malaysian perspective from a time of quarantine, lockdown and a new norm in the era of the pandemic akin to some of the international repositories that curate experiences during COVID-19.

Communication about health also comes from the aspect of literacy. There was a paucity of non-fictional or written narratives of COVID-19 experiences among the public in Malaysia. This is an area which can be improved on and encouraged. High-quality, internationally published books and narratives surrounding COVID-19 and health are easily accessible to Malaysians, particularly in this era of globalisation and electronic access. However, the benefits of these materials in the local context have yet to be determined and warrant further evaluation.

The health messages conveyed through digital arts in Malaysia were consistent with the trend of children’s educational channels worldwide advocating health precautionary measures. These approaches illustrated the importance of basic hygiene to young children in interesting ways as compared with the traditional verbal advice from parents.

Beyond animations, some of the works translated to meaningful work. For instance, Didi and Friends collaborated with MERCI Malaysia, UNICEF and the Government of Japan in distributing essential hygiene kits and health information to some of the most vulnerable populations in Malaysia. Furthermore, the partnerships between producers demonstrated their social responsibility in addressing the COVID-19 spread globally.

## Conclusion

In our views, the quantity and quality of arts produced in response to COVID-19 in Malaysia were encouraging and beneficial to the public. Communication of health messages such as raising awareness about COVID-19, reinforcement of prevention, precautionary measures, promoting adherence to standard operating procedures recommended, vaccination and instilling resilience were readily observed through the various arts. The implication of this landscape is that the public’s awareness of the perils of COVID-19 is raised, and members of the public are more informed about what they can do on their ends, and therefore are made more prepared to face the challenges ahead.

## Supplemental Material

sj-png-1-ped-10.1177_17579759221118256 – Supplemental material for Emerging stronger from COVID-19 through arts Click here for additional data file. Supplemental material, sj-png-1-ped-10.1177_17579759221118256 for Emerging stronger from COVID-19 through arts by Chong Yau Ong and Lee Wai Ching Deanna in Global Health Promotion

sj-png-2-ped-10.1177_17579759221118256 – Supplemental material for Emerging stronger from COVID-19 through arts Click here for additional data file. Supplemental material, sj-png-2-ped-10.1177_17579759221118256 for Emerging stronger from COVID-19 through arts by Chong Yau Ong and Lee Wai Ching Deanna in Global Health Promotion
