# Supplementary figures and images for: Emerging stronger from COVID-19 through arts
Source: Glob Health Promot. 2022 Sep 2;30(2):28–34. doi: 10.1177/17579759221118256 (PMC10273045; doi:10.1177/17579759221118256)

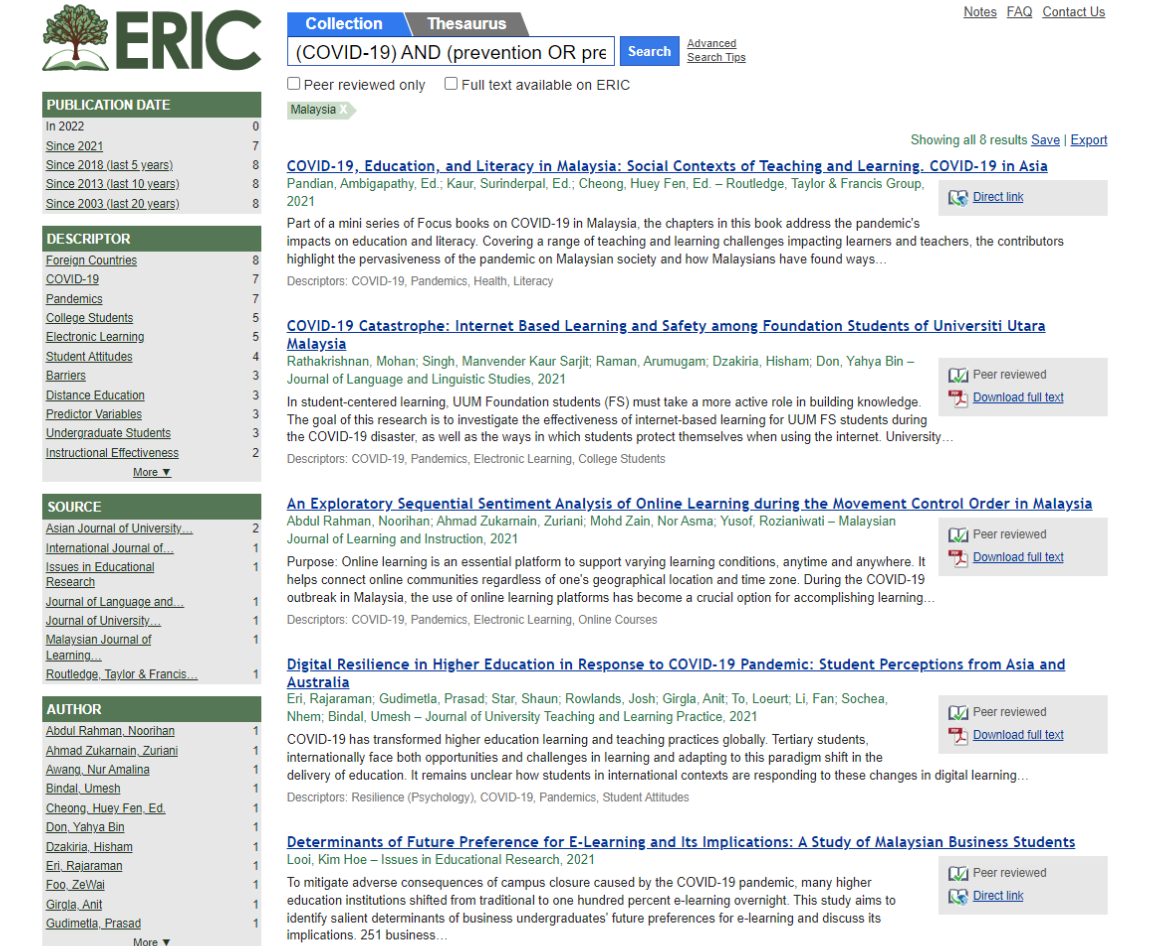

Supplement: sj-png-1-ped-10.1177_17579759221118256 – Supplemental material for Emerging stronger from COVID-19 through arts [file sj-png-1-ped-10.1177_17579759221118256.png]

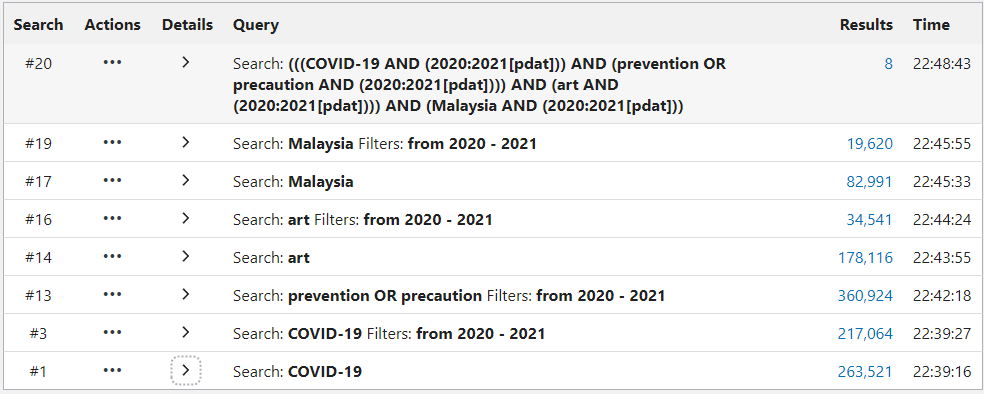

Supplement: sj-png-2-ped-10.1177_17579759221118256 – Supplemental material for Emerging stronger from COVID-19 through arts [file sj-png-2-ped-10.1177_17579759221118256.png]
